# Diet-induced obesity in zebrafish shares common pathophysiological pathways with mammalian obesity

**DOI:** 10.1186/1472-6793-10-21

**Published:** 2010-10-21

**Authors:** Takehiko Oka, Yuhei Nishimura, Liqing Zang, Minoru Hirano, Yasuhito Shimada, Zhipeng Wang, Noriko Umemoto, Junya Kuroyanagi, Norihiro Nishimura, Toshio Tanaka

**Affiliations:** 1 Department of Molecular and Cellular Pharmacology, Pharmacogenomics and Pharmacoinformatics, Mie University Graduate School of Medicine, 2-174 Edobashi, Tsu, Mie 514-8507, Japan; 2 Department of Bioinformatics, Mie University Life Science Research Center, 2-174 Edobashi, Tsu, Mie 514-8507, Japan; 3 Department of Medical Chemogenomics, Mie University Venture Business Laboratory, 2-174 Edobashi, Tsu, Mie 514-8507, Japan; 4 Mie University Medical Zebrafish Research Center, 2-174 Edobashi, Tsu, Mie 514-8507, Japan; 5 Department of Translational Medical Science, Mie University Graduate School of Medicine, 2-174 Edobashi, Tsu, Mie 514-8507, Japan

## Abstract

**Background:**

Obesity is a multifactorial disorder influenced by genetic and environmental factors. Animal models of obesity are required to help us understand the signaling pathways underlying this condition. Zebrafish possess many structural and functional similarities with humans and have been used to model various human diseases, including a genetic model of obesity. The purpose of this study was to establish a zebrafish model of diet-induced obesity (DIO).

**Results:**

Zebrafish were assigned into two dietary groups. One group of zebrafish was overfed with *Artemia *(60 mg dry weight/day/fish), a living prey consisting of a relatively high amount of fat. The other group of zebrafish was fed with *Artemia *sufficient to meet their energy requirements (5 mg dry weight/day/fish). Zebrafish were fed under these dietary protocols for 8 weeks. The zebrafish overfed with *Artemia *exhibited increased body mass index, which was calculated by dividing the body weight by the square of the body length, hypertriglyceridemia and hepatosteatosis, unlike the control zebrafish. Calorie restriction for 2 weeks was applied to zebrafish after the 8-week overfeeding period. The increased body weight and plasma triglyceride level were improved by calorie restriction. We also performed comparative transcriptome analysis of visceral adipose tissue from DIO zebrafish, DIO rats, DIO mice and obese humans. This analysis revealed that obese zebrafish and mammals share common pathophysiological pathways related to the coagulation cascade and lipid metabolism. Furthermore, several regulators were identified in zebrafish and mammals, including APOH, IL-6 and IL-1β in the coagulation cascade, and SREBF1, PPARα/γ, NR1H3 and LEP in lipid metabolism.

**Conclusion:**

We established a zebrafish model of DIO that shared common pathophysiological pathways with mammalian obesity. The DIO zebrafish can be used to identify putative pharmacological targets and to test novel drugs for the treatment of human obesity.

## Background

According to the World Health Organization, an estimated 310 million people worldwide are obese [1]. Such estimates are particularly alarming given the strong association between obesity and various adverse health consequences, including atherosclerosis, hypertension, type 2 diabetes and certain types of cancer [1-3]. While the exact causes remain elusive, it is now accepted that obesity is a complex, multifactorial disease that develops from an interaction between the genotype and the environment [1,2,4].

A number of genes involved in monogenic, syndromic and polygenic obesity have been identified [2,5]. In addition to genetic predisposition, environmental and behavioral factors resulting in increased physical inactivity and calorie intake also contribute to the development of obesity [6]. Numerous studies in rodents have attempted to characterize the functions of obesity-related genes and whole-animal responses to high-calorie and high-fat diets [7]. Such studies have generally shown that the obesity phenotype can differ based on the functions of knockout genes, genetic background, and dietary protocols [6-10]. This suggests the importance of developing and analyzing genetic and diet-induced models of obesity.

Although rodent models have greatly contributed to our understanding of human obesity [11], experiments using rodent models require considerable staff and infrastructural support, and are relatively expensive. Therefore, the development of simple and inexpensive animal models of obesity to complement the currently used rodent models has been anticipated. Recent studies on energy homeostasis in worms, fly and zebrafish have shown that these lower organisms can be used to unravel the metabolic processes underlying obesity [12,13].

As vertebrates, zebrafish possess many structural similarities with humans that worms and flies do not [13] and have been used to model various human diseases [14-19]. For example, zebrafish digestive organs, adipose tissues (AT), and skeletal muscle are physically arranged in a manner similar to their human counterparts [13]. Neural and endocrine signals regulating food intake are also conserved in zebrafish, including agouti-related protein (AgRP) [15,20], leptin [21] and adiponectin [22]. Although zebrafish larvae have been used in genetic and chemical screening experiments to identify novel genes involved in the regulation of energy homeostasis and potential therapeutic targets to treat obesity [13,23], it remains unclear whether zebrafish can be used as a model for diet-induced obesity (DIO), similar to that observed in mammals.

We have developed a zebrafish model for DIO and validated the model by several methods, including biochemical and histological analyses, diet therapies and DNA microarray analysis of visceral AT with comparison to those of mammalian obesity. These studies revealed that DIO zebrafish and obese mammals share common pathophysiological pathways, suggesting that zebrafish can be used as an alternative model of DIO.

## Results

### Zebrafish overfed with *Artemia *become obese

To develop a zebrafish model of DIO, we overfed the fish with freshly hatched nauplii of brine shrimp *Artemia*, a common food source for aquaculture [24]. Given the hierarchy of macronutrient effects on the perception of hunger (i.e. fat is least satiating while protein is most satiating [4]), it is plausible that zebrafish would overfeed on *Artemia *and become obese. Indeed, increases in the amount of dietary fat are associated with the risk of developing obesity [1,25].

We first assessed the consumption of *Artemia *by zebrafish. It was previously reported that the energy requirement for a Leopard *Danio*, a spotted color morph of zebrafish, is under 30 cal [26]. Therefore, we fed zebrafish at 3.5 months post-fertilization (mpf) with 5 or 60 mg of freshly hatched *Artemia *to meet their energy requirements (control) or to overfeed (OF), respectively. Zebrafish fed 5 or 60 mg of *Artemia *per day consumed about 80 or 50% of the provided *Artemia*, respectively (Methods). Because 1 mg of *Artemia *contains approximately 5 calories, it was estimated that a zebrafish would obtain 20 calories from 5 mg of *Artemia *at 80% consumption, and 150 calories from 60 mg of *Artemia *at 50% consumption. Zebrafish were fed under these dietary protocols for 8 weeks.

**Figure 1:**
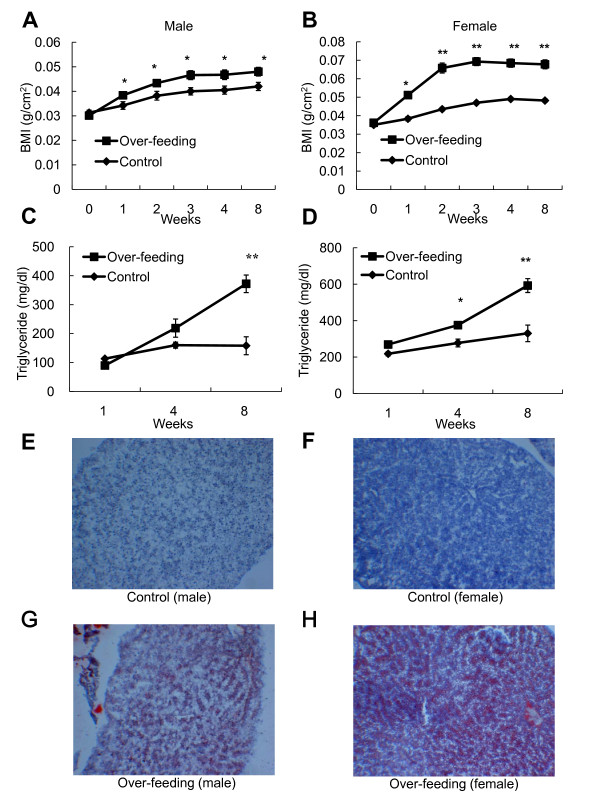
**Assessment of BMI, plasma TG and hepatic steatosis in zebrafish overfed with *Artemia***. Changes in BMI (g/cm^2^) in male (A) and female (B) zebrafish in the control and OF groups. Values are means ± SEM. OF group: n = 17 males and 16 females. Control group: n = 14 males and 17 females. Changes in plasma TG levels in male (C) and female (D) zebrafish in the control and OF groups. Values are means ± SEM. OF group: n = 7 males and 8 females. Control group: n = 7 males and 9 females. Statistical analyses were performed using Student's t-test to compare the OF and control groups at each time-point. *P < 0.05, **P < 0.01. (E-H) Oil Red O staining of liver sections from a male (E) and female (F) in the control group and a male (G) and female (H) in the OF group.

As shown in Figures 1A and 1B, significant (p < 0.05) increases in body mass index (BMI), which was calculated by dividing the body weight (g) by the square of the body length (cm), were evident in male (1.1-fold) and female (1.3-fold) zebrafish in the OF group compared with those in the control group within 1 week of the dietary protocol. This trend was maintained throughout the 8-week dietary protocol. As shown in Figures 1C and 1D, plasma TG levels were also significantly (p < 0.05) higher in male (2.4-fold) and female (1.8-fold) zebrafish in the OF group than in the control group at week 8, but not at week 1. Lipid-specific Oil Red O staining of frozen liver sections revealed abnormalities consistent with hepatosteatosis in both male (Figure 1G) and female (Figure 1H) zebrafish in the OF group at week 8, but not in the control group. These results indicate that zebrafish overfed with *Artemia *develop obesity in a manner similar to those observed in transgenic zebrafish overexpressing AgRP [15] and in mammalian models of DIO [8].

### Calorie restriction improved body weight and hypertriglyceridemia

**Figure 2:**
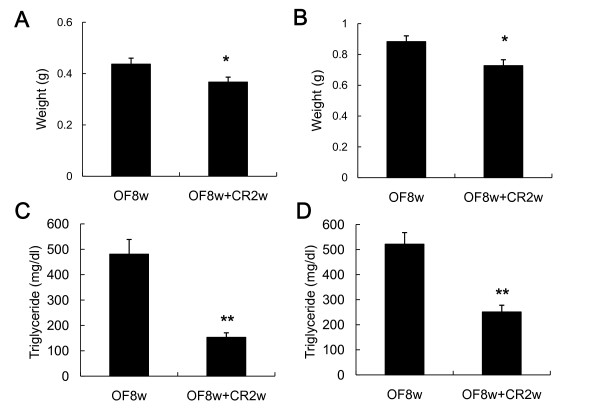
**Effects of calorie restriction on body weight and plasma TG level**. Body weight is significantly decreased by CR for 2 weeks in both male (A) and female (B) zebrafish. Values are means ± SEM. n = 7 for each group. The plasma TG level is significantly decreased by 2 weeks of CR in male (C) and female (D) zebrafish. Values are means ± SEM. n = 7 for each group. Statistical analyses were performed using Student's t-test to compare the OF8W and the OF8W+CR2W groups. *P < 0.05, **P < 0.01.

Calorie restriction (CR) is the most frequently prescribed treatment for obesity, although the health benefits of CR based on hard endpoints such as cardiovascular morbidity and mortality remain to be elucidated [1]. To validate the effect of CR, we fed the previously OF zebrafish with 2.5 mg of freshly hatched *Artemia *per day (50% of the control level) for 2 weeks (CR2W) after the 8-week overfeeding period (OF8W). The zebrafish consumed almost all of the provided *Artemia *(Methods) and the body weight of male and female zebrafish decreased to 84% and 82%, respectively, at OF8W (Figures 2A and 2B). Plasma TG levels were significantly (p < 0.05) decreased to 69% 52% at OF8W in male and female, respectively (Figures 2C and 2D). These data indicate that CR is effective for the treatment of DIO in zebrafish, similar to mammalian DIO [6-8].

### Gene expression profiling of visceral AT revealed common pathways in obesity

It has been reported that visceral AT plays an active role in the development of obesity-related complications such as cardiovascular diseases, insulin resistance and cancer [27]. Therefore, we performed DNA microarray analysis using Agilent Zebrafish DNA Oligoarrays of visceral AT from zebrafish overfed *Artemia *for 1 (OF1W) or 8 (OF8W) weeks, and treated the zebrafish with CR for 2 weeks (CR2W) after 8 weeks of overfeeding. The amount of visceral AT in the control group was so small that we could not extract total RNA. Therefore, we compared the genome-wide expression profiles of zebrafish at OF1W and OF8W to identify genes dysregulated by overfeeding using Rank Product Analysis (RankProd) [28]. RankProd analysis identified 120 and 48 genes as significantly (false discovery rate [FDR] <15%) increased and decreased, respectively, at OF8W [Additional file 1: Supplementary Table S1]. To identify the genes ameliorated by CR, we compared the genome-wide expression profiles of OF8W and CR2W. Among the 168 genes dysregulated by overfeeding, 97 genes (58%) were significantly (FDR <15%) ameliorated by CR [Additional file 2: Supplementary Table S2].

To compare the gene expression profiles of visceral AT of zebrafish and mammalian DIO, we downloaded multiple sets of microarray data from the Gene Expression Omnibus [29] (Table 1). GSE8700 was designed to analyze the differences in epididymal AT gene expression in rats fed a high-fat diet with those fed a control diet using Affymetrix Rat Genome Arrays [30]. GSE11790 was designed to analyze the differences in omental AT gene expression in mice fed a high-fat diet with those fed a control diet using custom arrays [31]. GSE15524 was designed to analyze the differences in omental AT gene expression in obese versus lean human individuals using CodeLink Uniset Human Bioarrays [32].

**Table 1:** Microarray datasets used for comparative transcriptome analysis

Dataset	Species	Adipose tissue	Control	Obesity
GSE18566	Zebrafish	Intra-abdominal	n = 4 (GSM461993-6)	n = 4 (GSM461997-2000)
GSE8700	Rat	Epididymal	n = 8 (GSM215579-86)	n = 7 (GSM215572-78)
GSE11790	Mouse	Omental	n = 4 (GSM298294-97)	n = 5 (GSM298289-93)
GSE15524	Human	Omental	n = 3 (GSM388904, 905, 930)	n = 11 (GSM388906-16)

We analyzed the microarray data using RankProd and identified genes that were differentially expressed in mammalian obesity. RankProd identified 461, 364 and 358 genes as being significantly (FDR <15%) dysregulated in rat, mouse and human obesity, respectively [Additional files 3, 4 and 5: Supplemental Tables S3,5]. We compared these gene lists with the 168 genes that were significantly dysregulated in zebrafish DIO [Additional file 6: Supplemental Table S6]. One gene (fatty acid binding protein 1, FABP1) was dysregulated in all three models of mammalian obesity, but was not spotted in the zebrafish microarray. Quantitative PCR (qPCR) analysis of zebrafish *fabp1a *revealed a positive trend in OF8W [Additional file 7: Supplemental Figure S1]. Fourteen genes were dysregulated in two models of mammalian obesity, while 22 genes were dysregulated in zebrafish DIO and in one model of mammalian obesity [Additional file 6: Supplemental Table S6]. qPCR analysis confirmed the induction of *hpx *in OF8W compared to OF1W [Additional file 7: Supplemental Figure S1].

Because the microarray platform differed among these four datasets, it is plausible that the composition of the gene list differs among these four species. Therefore, we analyzed the microarray data using Gene Set Enrichment Analysis (GSEA) [33] to identify gene sets defined based on prior biological knowledge, such as the Gene Ontology category. GSEA can determine whether members of a gene set belonging to a same biological pathway tend to occur toward the top of a given gene list, such as the gene list established from microarray analysis. The GSEA revealed that genes related to blood coagulation were significantly dysregulated in zebrafish, rat and mouse DIO and in human obesity. Genes related to platelet activation, fatty acid metabolism, cholesterol efflux, and triglyceride metabolism were significantly dysregulated in zebrafish, rat and mouse DIO (Table 2).

**Table 2:** Enriched biological processes showing dysregulation in zebrafish and mammalian obesity

	Zebrafish			Rat			Mouse			Human		
Process	NES	MC	p-value	NES	MC	p-value	NES	MC	p-value	NES	MC	p-value

Blood coagulation	2.3	3.1	0.0E+0	2.0	1.1	0.0E+0	1.7	1.1	9.5E-3	1.8	1.5	0.0E+0

Platelet activation	2.0	4.8	0.0E+0	1.8	1.1	6.1E-3	1.7	1.0	1.9E-2			

Fatty acid metabolism	2.2	1.6	0.0E+0	1.8	1.0	0.0E+0	1.8	1.0	4.5E-3			

Cholesterol efflux	1.6	1.1	0.0E+0	1.9	1.4	5.7E-3	1.8	1.3	0.0E+0			

Triglyceride metabolism	1.8	2.0	0.0E+0	1.8	1.2	5.8E-3	1.7	1.1	9.5E-3			

NES: normalized enrichment score; MC: median change in gene expression in the gene set

We also performed Sub-Network Enriched Analysis (SNEA) [34] to identify key molecules regulating the expression of the genes in the coagulation cascade or lipid metabolism identified by GSEA. SNEA revealed apolipoprotein H (APOH), interleukin-6 (IL-6) and interleukin-1β (IL-1β) as regulatory molecules in the coagulation cascade in obese zebrafish, rats, mice and humans (Table 3). SNEA also revealed sterol regulatory element binding transcription factor 1 (SREBF1), peroxisome proliferator-activated receptor alpha (PPARA), nuclear receptor subfamily 1 group H member 3 (NR1H3), PPAR gamma (PPARG) and leptin (LEP) as regulatory molecules involved in lipid metabolism in obese zebrafish, rats, mice and humans (Table 3).

**Table 3:** Regulatory factors involved in the coagulation cascade and lipid metabolism in zebrafish and mammalian obesity

Coagulation cascade	Zebrafish		Rat		Mouse		Human	
Regulatory factor	p-value	Rank^a^	p-value	Rank^a^	p-value	Rank^a^	p-value	Rank^a^

APOH	1.9E-08	1	8.4E-16	1	2.4E-12	2	9.1E-11	4

IL-6	4.6E-05	3	6.5E-10	5	8.2E-09	7	4.5E-10	6

IL-1β	7.0E-05	6	1.2E-08	8	1.6E-07	16	8.4E-09	9

								

Lipid metabolism	Zebrafish		Rat		Mouse		Human	

Regulatory factor	p-value	Rank^a^	p-value	Rank^a^	p-value	Rank^a^	p-value	Rank^a^

PPARα	1.6E-10	1	1.1E-23	1	9.1E-19	1	6.9E-24	1

PPARγ	1.7E-10	2	1.7E-15	3	2.1E-14	2	1.1E-16	3

NR1H3	1.0E-06	5	1.6E-15	2	2.7E-12	5	5.8E-17	2

LEP	1.8E-05	9	4.1E-10	13	3.7E-09	8	3.6E-12	10

SREBF1	1.8E-03	36	2.8E-13	7	5.7E-10	7	9.9E-10	15

^a^Regulatory factors are ranked based on the p-value in each dataset.

Regulatory factors ranked within the top 15 in at least three datasets are shown in this table.

**Figure 3:**
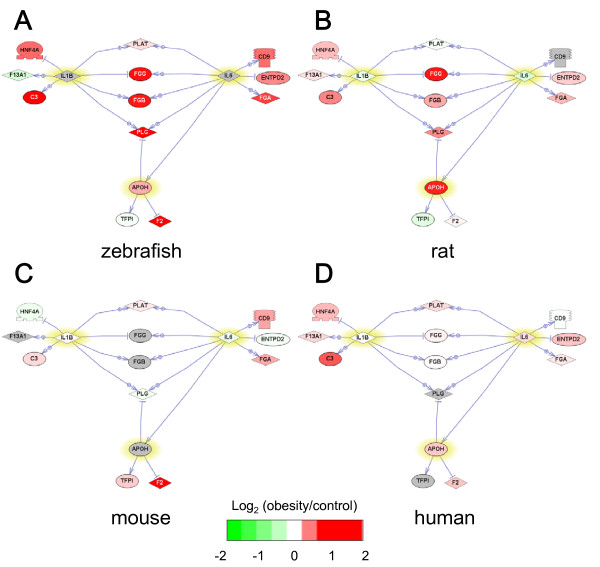
**Network for the coagulation cascade**. The network shows direct interactions between the three key regulatory molecules (IL-6, IL-1β and APOH, shown in yellow) identified by SNEA (Table 3) and their target genes in the coagulation cascade (blood coagulation and platelet activation) identified by GSEA (Table 2). Red and green denote genes with increased and decreased expression, respectively, in obese (OF8W) compared with control (OF1W) zebrafish. Gray denotes genes that were not spotted in the microarray. To reduce the complexity, the rat, mouse and human networks were constructed using the three regulatory factors and their target genes that were spotted in Agilent Zebrafish Oligoarray. A. zebrafish DIO (GSE18566), B. rat DIO (GSE8700), C. mouse DIO (GSE11790), D. human obesity (GSE15524).

**Figure 4:**
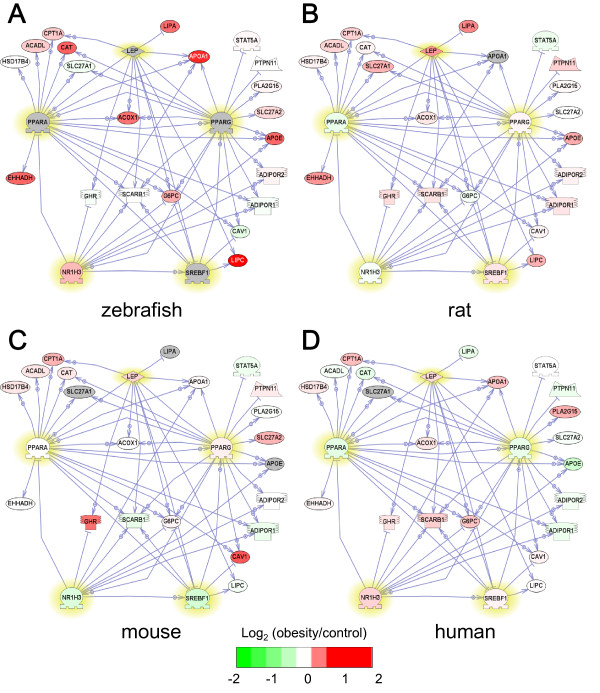
**Network for lipid metabolic pathways**. The network shows direct interactions between the five key regulatory molecules (SREBP1, PPARα/γ, NR3H1 and LEP, shown in yellow) identified by SNEA (Table 3) and their target genes involved in the lipid metabolic pathways (fatty acid metabolism, cholesterol efflux and triglyceride metabolism) identified by GSEA (Table 2). Red and green denote genes with increased and decreased expression, respectively, in obese (OF8W) compared with control (OF1W) zebrafish. Gray denotes genes that were not spotted in the microarray. To reduce the complexity, the rat, mouse and human networks were constructed using the five regulatory factors and their target genes that were spotted in the Agilent Zebrafish Oligoarray. A. zebrafish DIO (GSE18566), B. rat DIO (GSE8700), C. mouse DIO (GSE11790), D. human obesity (GSE15524).

**Figure 5:**
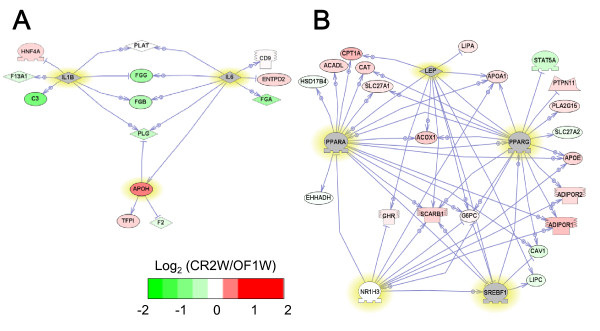
**Effects of CR on the coagulation cascade and lipid metabolism in DIO zebrafish**. A. Network showing direct interactions between the three key regulatory molecules (IL-6, IL-1β and APOH, shown in yellow) identified by SNEA (Table 3) and their target genes in the coagulation cascade (blood coagulation and platelet activation) identified by GSEA (Table 2). B. Network showing direct interactions between the five key regulatory molecules (SREBP1, PPARα/γ, NR3H1 and LEP, shown in yellow) identified by SNEA (Table 3) and their target genes involved in the lipid metabolic pathways (fatty acid metabolism, cholesterol efflux and triglyceride metabolism) identified by GSEA (Table 2). Red and green denote genes with increased and decreased expression, respectively, at CR2W compared with OF1W. Gray denotes genes that were not spotted in the microarray.

The interactions between these regulatory molecules and their target genes are shown in Figure 3 for the coagulation cascade and Figure 4 for lipid metabolism. The color of many genes, including fibrinogen-γ (FGG) and factor II (F2, also known as thrombin) in Figure 3 and apolipoprotein A-I (APOA1) in Figure 4, are red, suggesting that genes related to coagulation cascade and lipid metabolism are induced by obesity. By contrast, the color of these genes was turned green by CR, suggesting that CR ameliorates dysfunctions in the coagulation cascade and lipid metabolism (Figure 5). These results suggest that DIO zebrafish share common pathophysiological pathways with mammalian obesity.

## Discussion

In this study, we developed a zebrafish model of DIO. DIO zebrafish exhibited increased BMI, hypertriglyceridemia and hepatosteatosis. Comparative transcriptome analysis using visceral AT revealed that the DIO zebrafish and obese mammals share common pathophysiological pathways. These findings suggest that the DIO zebrafish model can be used to identify putative pharmacological targets and to test novel drugs for the treatment of human obesity.

### Zebrafish can be used as a useful animal model of DIO

The regulation of feeding behavior occurs via close interactions between peripheral regions and the brain [1-4,35-38]. Peripheral endocrine and metabolic factors convey information regarding nutritional status to the brain. The peripheral signals include satiety signals, such as PYY [39], GLP-1 [40] and ghrelin [41], which originate from the gastrointestinal tract, as well as adiposity signals, such as adiponectin [22] and insulin [42], the blood levels of which are proportional to body nutrient stores [38]. The brain then processes this peripheral information and induces neuropeptide signaling (for example, via NPY [43], α-MSH [20], AgRP [20] and orexin [44]), mainly from the hypothalamus, to stimulate or inhibit feeding. These peripheral and central factors controlling food intake are well conserved in zebrafish [20,22,39-45].

Probably because of the low satiating effect of fat consumption [4], high dietary fat intake is associated with an increased risk of obesity [1,25]. Because of the relatively high fat content in *Artemia*, as compared with flake foods, we fed the zebrafish a diet consisting solely of *Artemia *to induce obesity. We have demonstrated that zebrafish overfed with *Artemia *showed significant increases in BMI and plasma TG levels and hepatosteatosis, consistent with obesity observed in humans and rodent models of DIO [1,6,7]. Of note, there seemed to be no decrease in physical activity in the zebrafish overfed *Artemia *(data not shown), indicating that the development of obesity is most likely to be a result of the increased intake of a high-fat diet.

There are several advantages to the DIO zebrafish model. First, zebrafish (*AB *line) respond well to the *Artemia *diet because almost all of the zebrafish overfed *Artemia *developed obesity. The C57BL/6J (B6) line has been widely used for DIO in mice because these mice are very susceptible to obesity when fed a high-fat diet [6-9]. However, there are variations in adiposity among individual B6 mice [46,47]. Similarly, when outbred Sprague-Dawley rats are fed a high-fat diet, about half become obese, while the other half are resistant to DIO [6-10]. The relatively homogenous responses of zebrafish to overfeeding with *Artemia *suggest that these fish represent an excellent alternative model species for experimental research on DIO. Furthermore, the dietary protocol to induce obesity in zebrafish is simple and can be applied to other zebrafish lines. For example, we are currently applying the protocol to the *Casper *line, which have transparent abdomens, even in the adult stage [48]. Using DIO *Casper *zebrafish, visceral AT can be visualized in a living animal under a fluorescent microscope by staining the AT with a fluorescent dye, such as Nile Red [49]. This feature makes it possible to monitor the short- and long-term effects of a therapeutic intervention on the amount of visceral AT in live DIO zebrafish. Finally, zebrafish are small and easy to maintain in large stocks because of their high fecundity, thus making zebrafish amenable for medium-to-high throughput screening for early drug discovery [50].

One limitation of the zebrafish DIO model is the apparent absence of brown AT [51]. The development of obesity in mammals not only depends on the balance between food intake and calorie utilization, but also on the balance between white AT and brown AT [51]. Therefore, zebrafish DIO may not be suitable to identify signaling pathways related to brown AT.

### Comparative transcriptomics of visceral AT revealed common pathophysiological pathways in zebrafish and mammalian obesity

Genome-wide expression assays using DNA microarrays allow rapid screening and quantification of differences in large groups of functionally related genes and are thus well-suited to studies of pathways dysregulated in obesity [52]. We compared the visceral AT expression profiles of zebrafish, rat, mouse and human obesity. The comparative transcriptome analysis revealed that several genes involved in blood coagulation, platelet activation, fatty acid metabolism, cholesterol efflux, and triglyceride metabolism were dysregulated in both zebrafish and mammalian obesity. IL-6, IL-1β and APOH were identified as regulatory factors involved in blood coagulation and platelet activation in zebrafish and mammalian obesity. Similarly, SREBP1, PPARα/γ, NR3H1 and LEP were identified as common regulatory factors for fatty acid metabolism, cholesterol efflux and triglyceride metabolism in zebrafish and mammalian obesity.

Visceral AT dysfunction can play a causal role in the prothrombotic state observed in obesity by affecting hemostasis, coagulation and fibrinolysis [53-55]. It has also been shown that IL-6 and IL-1β, secreted from visceral AT, induce the biosynthesis of fibrinogen from visceral AT and liver. Fibrinogen is a substrate of F2 (thrombin) in the final step of the coagulation cascade, and its presence is essential for platelet aggregation [53]. Of interest, plasma fibrinogen levels were found to be significantly higher in obese subjects than in age- and sex-matched non-obese individuals [53]. Significant correlations have been reported between fibrinogen and BMI and the waist-to-hip ratio [53]. It has also been reported that substantial weight loss reduces fibrinogen levels more effectively than modest weight reduction [53]. Consistent with these reports, OF and CR significantly induced and reduced, respectively, the expression of fibrinogen in zebrafish (Figures 3A and 5A, Additional files 1 and 2: Supplemental Table S1 and S2). APOH binds to activated protein C (APC), an anticoagulant enzyme that is activated by activation of the protein C zymogen by the thrombin-thrombomodulin complex on the surface of endothelial cells, platelets and monocytes [56]. However, the effect of APOH binding to APC is inconclusive and the functional role of APOH in obesity remains to be elucidated.

Dyslipidemia is commonly seen in obesity [57,58] and is characterized by an increased flux of free fatty acids, elevated TG levels, low high-density lipoprotein cholesterol levels and increased low-density lipoprotein levels [57]. Fatty acid metabolism, cholesterol efflux and triglyceride metabolism are closely related to these pathways. This study revealed that SREBP1, PPARα/γ, NR3H1 and LEP are key regulatory factors in these pathways and are expressed in zebrafish and mammalian obesity. PPARs mediate adaptive metabolic responses to increased systemic lipid availability and are activated by endogenous or dietary lipids [59]. PPARα promotes lipid clearance by increasing tissue fat oxidization [59,60] while PPARγ promotes lipid storage in white AT, as well as preadipocyte differentiation to mature adipocytes [59]. SREBP1 is an important transcription factor that regulates the transcription of many lipid genes and participates in adipocyte differentiation by stimulating PPARγ [61,62]. NR3H1, also known as liver X receptor A (LXRA), has been shown to regulate lipid and carbohydrate homeostasis [63]. LEP, an adiposity hormone produced by white AT, reflects total fat mass [64]. Although we did not detect a statistically significant difference between OF8W and OF1W in terms of the mRNA expression level of *lep *measured by qPCR (data not shown), the expression of *apoa1*, a transcriptional target gene of leptin [65], was significantly induced and reduced by OF and CR, respectively (Figures 4A and 5B) [Additional files 1, 2 and 7: Supplemental Tables S1 and S2, and Figure S1], suggesting that leptin protein levels were likely to be increased in zebrafish DIO. It is noteworthy that the functional importance of SREBP1, PPARα/γ, NR3H1 and LEP in obesity has been shown in many genetic studies [2,11,63,66].

## Conclusions

Here, we have shown that the DIO zebrafish model shares common pathophysiological pathways with mammalian obesity and can be used to identify putative pharmacological targets of human obesity. For example, we are currently performing genome-wide expression profiling of the DIO zebrafish liver. The zebrafish model can also be used to study the hypothalamic-pituitary axis, the main link between the central nervous and the endocrine system [67]. Further studies are needed to examine whether complications related to obesity such as insulin resistance, cardiovascular diseases and cancer can be studied in DIO zebrafish.

## Methods

### Feeding zebrafish

Adult zebrafish (*AB *line, ZIRC, Eugene, OR, USA) were kept at 28°C under a 14-h light:10-h dark cycle, and water conditions of environmental quality were maintained according to an established protocol [68]. Zebrafish at 3.5 mpf were assigned into two dietary groups (overfeeding and maintenance groups) with approximately five fish per 2-L tank. Zebrafish in the overfeeding group were fed three times per day with freshly hatched *Artemia *(corresponding to 60 mg cysts/fish/day). Zebrafish in the maintenance group were fed freshly hatched *Artemia *(corresponding to 5 mg cysts/fish/day) once per day. For calorie restriction, the zebrafish were fed freshly hatched *Artemia *(corresponding to 2.5 mg cysts/fish/day) for 2 weeks after being overfed for 8 weeks. Commercial flake food (Hikari Tropical Fancy Guppy, Kyorin, Hyogo, Japan) consists of 9% fat, 17% carbohydrate and 59% protein, whereas *Artemia *nauplii consist of 22% fat, 16% carbohydrate and 44% protein (dry weight basis).

The percentage of consumed *Artemia *was estimated by counting *Artemia *before and after feeding. Briefly, 1 mL of water was collected from 1700 mL of water containing freshly hatched *Artemia *corresponding to 100 or 8.5 mg cysts. The numbers of hatched *Artemia *were counted three times to determine a mean count. After counting, the samples were returned to the 2-L tank. Then, five fish were transferred to the tank for feeding. After 2 hours, the number of *Artemia *was counted as before feeding. Zebrafish fed 5 or 60 mg of *Artemia *per day consumed about 80 or 50% of the provided *Artemia*, respectively. Zebrafish fed 2.5 mg of *Artemia *per day consumed almost all of the provided *Artemia*.

### Measurement of BMI and plasma TG

The body weight and length of zebrafish were measured weekly throughout the study. Zebrafish length was measured from the head to the end of the body. Blood was withdrawn from the dorsal artery of the zebrafish at the indicated times after an overnight fast to measure plasma TG (TG L-type assay, Wako, Tokyo, Japan).

### Oil Red O staining

Zebrafish were fixed using 4% formaldehyde solution in PBS. The fixed samples were rapidly frozen in liquid nitrogen-cooled isopentane, embedded in Tissue-Tek (Sakura Finetek Europe, Zoeterwoude, Netherlands) and cut using a cryostat. After drying, the sections were fixed in 4% formalin and rinsed with distilled water. The sections were then immersed in a working solution of Oil Red O (Wako) for 15 min and rinsed with distilled water. Sections were also counterstained using Mayer's hematoxylin to visualize the nuclei.

### Nile Red staining

A stock solution of Nile Red (Wako) was prepared by dissolving 10 mg of Nile Red in 10 mL acetone. Zebrafish were transferred into 1000 mL of swimming water containing 100 μg of Nile Red and incubated overnight in the dark at 28°C. After incubation, the fish were rinsed in fresh water and observed with a fluorescence microscope (MZ 16F, Leica, Tokyo, Japan) using a GFP2 filter (Leica).

### DNA microarray analysis of visceral AT of DIO zebrafish

The visceral (omental) AT of male DIO zebrafish stained with Nile Red was collected by surgical extraction under a fluorescence microscope. The AT was stored in RNA-later (Applied Biosystems, Foster City, CA, USA). Total RNA was then extracted using an RNeasy Mini Kit (Qiagen, Valencia, CA, USA), qualified by an Agilent Bioanalyzer 2100 (Agilent, Santa Clara, CA, USA) and quantified using a spectrophotometer (NanoDrop ND-100, Wilmington, DE, USA). Three hundred nanograms of total RNA from each visceral AT depot were converted into labeled cRNA using the Low RNA Input Fluorescent Linear Amplification Kit (Agilent). Cy3-labeled cRNA (1.5 μg) was hybridized to Agilent Zebrafish Whole Genome Oligo Microarrays (G2518A) according to the manufacturer's protocol. The microarray has 22,000 different probes. Each probe is spotted twice distantly in a microarray. The hybridized microarrays were scanned (Agilent G2565BA) and analyzed using Feature Extraction software (Agilent). The data were normalized according to the manufacturer's protocol (Agilent). RankProd analysis [28] was performed using Bioconductor [69] to identify differentially expressed genes between two groups by calculating the FDR. Differentially expressed genes (FDR <15% for both duplicated probes) were then converted to human orthologs using the Life Science Knowledge Bank (World Fusion, Tokyo, Japan). The gene symbols of human orthologs were used for functional analysis.

### DNA microarray analysis of visceral AT in mammalian obesity

The microarray data ([GEO:GDS8700] [30], [GEO:GSE11790] [31] and [GEO;GSE15524] [32]) were downloaded from Gene Expression Omnibus [29]. We used microarray data that were filtered and normalized and applied the data to RankProd analysis [28] to identify differentially expressed genes between obese and control mammals. The conversion to human orthologs and functional analysis were performed as described above for visceral AT from DIO zebrafish. The microarray data of zebrafish DIO have been deposited in NCBI GEO and can be downloaded from GEO main page (http://www.ncbi.nlm.nih.gov/geo/) with accession number GSE18566. GSEA and SNEA were performed using Pathway Studio 7 (Ariadne Genomics, Rockville, MD, USA). Pathway Studio is a program used to visualize and analyze biological pathways and gene regulation networks. This program includes the ResNet database of more than 500,000 functional relationships and the MedScan tool for automatic extraction of information from the scientific literature. Pathway Studio 7 draws networks based on the extracted information and was used to draw Figures 3, 4 and 5.

GSEA was used to determine the list of biological pathways dysregulated in obesity using the Kolmogorov-Smirnov enrichment algorithm with 400 random permutations to determine statistical significance. SNEA was used to identify molecules regulating expression of genes in the coagulation cascade (blood coagulation and platelet activation) and in lipid metabolism (fatty acid metabolism, cholesterol efflux and triglyceride metabolism), both of which were identified by GSEA to be dysregulated in zebrafish and mammalian obesity. SNEA built sub-networks of genes related to the coagulation cascade or the lipid metabolism and identified the central "seed" of each network prioritized by the p-value based on the criterion "Expression Targets" in the ResNet database.

### qPCR analysis

Total RNAs from zebrafish visceral AT were used to generate cDNAs using an iScript Select cDNA Synthesis Kit (Bio-Rad, Hercules, CA, USA). qPCR was done using an ABI Prism 7300 (Life Technologies, Carlsbad, CA, USA) with SYBR Green Realtime PCR Master Mix Plus (Toyobo, Osaka, Japan). The thermal cycling condition comprised an initial step at 95°C for 1 min followed by 40 cycles of 95°C for 15 sec, 60°C for 15 sec and 72°C for 45 sec. The primers used in this study are shown in [Additional file 8: Supplementary Table S7]. Data were normalized by the quantity of glyceraldehyde-3-phosphate dehydrogenase (*gapdh*). This allowed us to account for any variability in the initial template concentration as well as the conversion efficiency of the reverse transcription reaction.

### Statistical Analysis

Results are expressed as means ± SEM. Differences between groups were tested for statistical significance using Student's t-test.

## Authors' contributions

TO carried out the experiments and helped to draft the manuscript. YN carried out qPCR experiments and drafted the manuscript. LZ carried out the assessment of feeding. MH participated in the design of the study. YS helped to draft the manuscript. ZW, NU and JK helped to carry out the experiments. NN conceived the study. TT conceived the study and drafted the manuscript. All authors read and approved the final manuscript.

## Supplementary Material

Additional file 1 **Supplemental Table S1**. Genes dysregulated in DIO zebrafish (OF8W vs OF1W) Click here for file

Additional file 2 **Supplemental Table S2**. Genes ameliorated by CR in zebrafish DIO (CR2W vs OF8W) Click here for file

Additional file 3 **Supplemental Table S3**. Genes dysregulated in DIO rats (GSE8700) Click here for file

Additional file 4 **Supplemental Table S4**. Genes dysregulated in DIO mice (GSE11790) Click here for file

Additional file 5 **Supplemental Table S5**. Genes dysregulated in obese humans (GSE15524) Click here for file

Additional file 6 **Supplemental Table S6**. Genes differentially expressed in visceral AT in the same direction in at least two obesity models Click here for file

Additional file 7 **Supplemental Figure S1**. Validation of the differential gene expression by qPCR analysis. Total RNA was extracted from zebrafish visceral AT and qPCR analysis was performed to validate the differential expression identified by microarray analysis. Results represent means ± S.E.M. of each group. P-value was calculated by Student's t-test using OF1W (N = 6) vs. OF8W (N = 4). *p < 0.05. Click here for file

Additional file 8 **Supplemental Table S7**. Primers used in qPCR analysis Click here for file

## References

[B1] Bessesen DH Update on obesity J Clin Endocrinol Metab 2008 93 6 2027 2034 10.1210/jc.2008-0520 18539769

[B2] Bell CG Walley AJ Froguel P The genetics of human obesity Nat Rev Genet 2005 6 3 221 234 10.1038/nrg1556 15703762

[B3] Adan RA Vanderschuren LJ ElF S Anti-obesity drugs and neural circuits of feeding Trends Pharmacol Sci 2008 29 4 208 217 10.1016/j.tips.2008.01.008 18353447

[B4] Speakman JR Obesity: the integrated roles of environment and genetics J Nutr 2004 134 8 Suppl 2090S 2105S 15284410 10.1093/jn/134.8.2090S

[B5] Rankinen T Zuberi A Chagnon YC Weisnagel SJ Argyropoulos G Walts B Perusse L Bouchard C The human obesity gene map: the 2005 update Obesity (Silver Spring) 2006 14 4 529 644 10.1038/oby.2006.71 16741264

[B6] Casper RC Sullivan EL Tecott L Relevance of animal models to human eating disorders and obesity Psychopharmacology (Berl) 2008 199 3 313 329 10.1007/s00213-008-1102-2 18317734

[B7] Speakman J Hambly C Mitchell S Krol E Animal models of obesity Obes Rev 2007 8 Suppl 1 55 61 10.1111/j.1467-789X.2007.00319.x 17316303

[B8] West DB York B Dietary fat, genetic predisposition, and obesity: lessons from animal models Am J Clin Nutr 1998 67 3 Suppl 505S 512S 9497161 10.1093/ajcn/67.3.505S

[B9] Buettner R Scholmerich J Bollheimer LC High-fat diets: modeling the metabolic disorders of human obesity in rodents Obesity (Silver Spring) 2007 15 4 798 808 10.1038/oby.2007.608 17426312

[B10] Butler AA Cone RD The melanocortin receptors: lessons from knockout models Neuropeptides 2002 36 2-3 77 84 10.1054/npep.2002.0890 12359499

[B11] Powell DR Obesity drugs and their targets: correlation of mouse knockout phenotypes with drug effects in vivo Obes Rev 2006 7 1 89 108 10.1111/j.1467-789X.2006.00220.x 16436105

[B12] Chiang SH MacDougald OA Will fatty worms help cure human obesity? Trends Genet 2003 19 10 523 525 10.1016/j.tig.2003.08.002 14550624

[B13] Schlegel A Stainier DY Lessons from "lower" organisms: what worms, flies, and zebrafish can teach us about human energy metabolism PLoS Genet 2007 3 11 e199 10.1371/journal.pgen.0030199 18081423 PMC2098794

[B14] Lieschke GJ Currie PD Animal models of human disease: zebrafish swim into view Nat Rev Genet 2007 8 5 353 367 10.1038/nrg2091 17440532

[B15] Song Y Cone RD Creation of a genetic model of obesity in a teleost FASEB J 2007 21 9 2042 2049 10.1096/fj.06-7503com 17341684

[B16] Tanaka T Oka T Shimada Y Umemoto N Kuroyanagi J Sakamoto C Zang L Wang Z Nishimura Y Pharmacogenomics of cardiovascular pharmacology: pharmacogenomic network of cardiovascular disease models J Pharmacol Sci 2008 107 1 8 14 10.1254/jphs.08R03FM 18490853

[B17] Kinkel MD Prince VE On the diabetic menu: Zebrafish as a model for pancreas development and function Bioessays 2009 31 2 139 152 10.1002/bies.200800123 19204986 PMC2770330

[B18] Wang Z Nishimura Y Shimada Y Umemoto N Hirano M Zang L Oka T Sakamoto C Kuroyanagi J Tanaka T Zebrafish beta-adrenergic receptor mRNA expression and control of pigmentation Gene 2009 446 1 18 27 10.1016/j.gene.2009.06.005 19540320

[B19] Watanabe K Nishimura Y Oka T Nomoto T Kon T Shintou T Hirano M Shimada Y Umemoto N Kuroyanagi J In vivo imaging of zebrafish retinal cells using fluorescent coumarin derivatives BMC Neurosci 2010 11 1 116 10.1186/1471-2202-11-116 20843315 PMC2945357

[B20] Song Y Golling G Thacker TL Cone RD Agouti-related protein (AGRP) is conserved and regulated by metabolic state in the zebrafish, Danio rerio Endocrine 2003 22 3 257 265 10.1385/ENDO:22:3:257 14709799

[B21] Gorissen M Bernier NJ Nabuurs SB Flik G Huising MO Two divergent leptin paralogues in zebrafish (Danio rerio) that originate early in teleostean evolution J Endocrinol 2009 201 3 329 339 10.1677/JOE-09-0034 19293295

[B22] Nishio S Gibert Y Bernard L Brunet F Triqueneaux G Laudet V Adiponectin and adiponectin receptor genes are coexpressed during zebrafish embryogenesis and regulated by food deprivation Dev Dyn 2008 237 6 1682 1690 10.1002/dvdy.21559 18489000

[B23] Jones KS Alimov AP Rilo HL Jandacek RJ Woollett LA Penberthy WT A high throughput live transparent animal bioassay to identify non-toxic small molecules or genes that regulate vertebrate fat metabolism for obesity drug development Nutr Metab (Lond) 2008 5 23 10.1186/1743-7075-5-23 18752667 PMC2531115

[B24] Bengtson DA Leger P Sorgeloos P Use of artemia as a food source for aquaculture 1991 29 CRC Press

[B25] Moussavi N Gavino V Receveur O Could the quality of dietary fat, and not just its quantity, be related to risk of obesity? Obesity (Silver Spring) 2008 16 1 7 15 10.1038/oby.2007.14 18223605

[B26] Pannevis MC Earle KE Maintenance energy requirement of five popular species of ornamental fish J Nutr 1994 124 12 Suppl 2616S 2618S 7996251 10.1093/jn/124.suppl_12.2616S

[B27] Ahima RS Adipose tissue as an endocrine organ Obesity (Silver Spring) 2006 14 Suppl 5 242S 249S 10.1038/oby.2006.317 17021375

[B28] Breitling R Armengaud P Amtmann A Herzyk P Rank products: a simple, yet powerful, new method to detect differentially regulated genes in replicated microarray experiments FEBS Lett 2004 573 1-3 83 92 10.1016/j.febslet.2004.07.055 15327980

[B29] Barrett T Troup DB Wilhite SE Ledoux P Rudnev D Evangelista C Kim IF Soboleva A Tomashevsky M Marshall KA NCBI GEO: archive for high-throughput functional genomic data Nucleic Acids Res 2009 37 Database D885 890 10.1093/nar/gkn764 18940857 PMC2686538

[B30] Li S Zhang HY Hu CC Lawrence F Gallagher KE Surapaneni A Estrem ST Calley JN Varga G Dow ER Assessment of diet-induced obese rats as an obesity model by comparative functional genomics Obesity (Silver Spring) 2008 16 4 811 818 10.1038/oby.2007.116 18239588

[B31] Poussin C Hall D Minehira K Galzin AM Tarussio D Thorens B Different transcriptional control of metabolism and extracellular matrix in visceral and subcutaneous fat of obese and rimonabant treated mice PLoS One 2008 3 10 e3385 10.1371/journal.pone.0003385 19030233 PMC2586343

[B32] MacLaren RE Cui W Lu H Simard S Cianflone K Association of adipocyte genes with ASP expression: a microarray analysis of subcutaneous and omental adipose tissue in morbidly obese subjects BMC Med Genomics 2010 3 3 10.1186/1755-8794-3-3 20105310 PMC2843642

[B33] Subramanian A Tamayo P Mootha VK Mukherjee S Ebert BL Gillette MA Paulovich A Pomeroy SL Golub TR Lander ES Gene set enrichment analysis: a knowledge-based approach for interpreting genome-wide expression profiles Proc Natl Acad Sci USA 2005 102 43 15545 15550 10.1073/pnas.0506580102 16199517 PMC1239896

[B34] Kotelnikova E Yuryev A Mazo I Daraselia N Computational approaches for drug repositioning and combination therapy design J Bioinform Comput Biol 2010 8 3 593 606 10.1142/S0219720010004732 20556864

[B35] Badman MK Flier JS The gut and energy balance: visceral allies in the obesity wars Science 2005 307 5717 1909 1914 10.1126/science.1109951 15790843

[B36] Spiegelman BM Flier JS Obesity and the regulation of energy balance Cell 2001 104 4 531 543 10.1016/S0092-8674(01)00240-9 11239410

[B37] Cummings DE Overduin J Gastrointestinal regulation of food intake J Clin Invest 2007 117 1 13 23 10.1172/JCI30227 17200702 PMC1716217

[B38] Volkoff H The role of neuropeptide Y, orexins, cocaine and amphetamine-related transcript, cholecystokinin, amylin and leptin in the regulation of feeding in fish Comp Biochem Physiol A Mol Integr Physiol 2006 144 3 325 331 10.1016/j.cbpa.2005.10.026 16326123

[B39] Amores A Force A Yan YL Joly L Amemiya C Fritz A Ho RK Langeland J Prince V Wang YL Zebrafish hox clusters and vertebrate genome evolution Science 1998 282 5394 1711 1714 10.1126/science.282.5394.1711 9831563

[B40] Mommsen TP Mojsov S Glucagon-like peptide-1 activates the adenylyl cyclase system in rockfish enterocytes and brain membranes Comp Biochem Physiol B Biochem Mol Biol 1998 121 1 49 56 10.1016/S0305-0491(98)10110-4 9972283

[B41] Amole N Unniappan S Fasting induces preproghrelin mRNA expression in the brain and gut of zebrafish, Danio rerio Gen Comp Endocrinol 2009 161 1 133 7 10.1016/j.ygcen.2008.11.002 19027742

[B42] Huang H Vogel SS Liu N Melton DA Lin S Analysis of pancreatic development in living transgenic zebrafish embryos Mol Cell Endocrinol 2001 177 1-2 117 124 10.1016/S0303-7207(01)00408-7 11377827

[B43] Mathieu M Trombino S Argenton F Larhammar D Vallarino M Developmental expression of NPY/PYY receptors zYb and zYc in zebrafish Ann N Y Acad Sci 2005 1040 399 401 10.1196/annals.1327.073 15891072

[B44] Novak CM Jiang X Wang C Teske JA Kotz CM Levine JA Caloric restriction and physical activity in zebrafish (Danio rerio) Neurosci Lett 2005 383 1-2 99 104 10.1016/j.neulet.2005.03.048 15936519

[B45] Volkoff H Peter RE Feeding behavior of fish and its control Zebrafish 2006 3 2 131 140 10.1089/zeb.2006.3.131 18248256

[B46] Burcelin R Crivelli V Dacosta A Roy-Tirelli A Thorens B Heterogeneous metabolic adaptation of C57BL/6J mice to high-fat diet Am J Physiol Endocrinol Metab 2002 282 4 E834 842 11882503 10.1152/ajpendo.00332.2001

[B47] Koza RA Nikonova L Hogan J Rim JS Mendoza T Faulk C Skaf J Kozak LP Changes in gene expression foreshadow diet-induced obesity in genetically identical mice PLoS Genet 2006 2 5 e81 10.1371/journal.pgen.0020081 16733553 PMC1464831

[B48] White RM Sessa A Burke C Bowman T LeBlanc J Ceol C Bourque C Dovey M Goessling W Burns CE Transparent adult zebrafish as a tool for in vivo transplantation analysis Cell Stem Cell 2008 2 2 183 189 10.1016/j.stem.2007.11.002 18371439 PMC2292119

[B49] Greenspan P Mayer EP Fowler SD Nile red: a selective fluorescent stain for intracellular lipid droplets J Cell Biol 1985 100 3 965 973 10.1083/jcb.100.3.965 3972906 PMC2113505

[B50] Barros TP Alderton WK Reynolds HM Roach AG Berghmans S Zebrafish: an emerging technology for in vivo pharmacological assessment to identify potential safety liabilities in early drug discovery Br J Pharmacol 2008 154 7 1400 1413 10.1038/bjp.2008.249 18552866 PMC2492106

[B51] Gesta S Tseng YH Kahn CR Developmental origin of fat: tracking obesity to its source Cell 2007 131 2 242 256 10.1016/j.cell.2007.10.004 17956727

[B52] Nadler ST Attie AD Please pass the chips: genomic insights into obesity and diabetes J Nutr 2001 131 8 2078 2081 11481397 10.1093/jn/131.8.2078

[B53] Mertens I Van Gaal LF Obesity, haemostasis and the fibrinolytic system Obes Rev 2002 3 2 85 101 10.1046/j.1467-789X.2002.00056.x 12120424

[B54] Nieuwdorp M Stroes ES Meijers JC Buller H Hypercoagulability in the metabolic syndrome Curr Opin Pharmacol 2005 5 2 155 159 10.1016/j.coph.2004.10.003 15780824

[B55] Faber DR de Groot PG Visseren FL Role of adipose tissue in haemostasis, coagulation and fibrinolysis Obes Rev 2009 10 5 554 563 10.1111/j.1467-789X.2009.00593.x 19460118

[B56] Sola E Navarro S Medina P Vaya A Estelles A Hernandez-Mijares A Espana F Activated protein C levels in obesity and weight loss influence Thromb Res 2009 123 5 697 700 10.1016/j.thromres.2008.07.017 18834618

[B57] Kolovou GD Anagnostopoulou KK Cokkinos DV Pathophysiology of dyslipidaemia in the metabolic syndrome Postgrad Med J 2005 81 956 358 366 10.1136/pgmj.2004.025601 15937200 PMC1743285

[B58] Unger RH Clark GO Scherer PE Orci L Lipid homeostasis, lipotoxicity and the metabolic syndrome Biochim Biophys Acta 2010 1801 3 209 214 19948243 10.1016/j.bbalip.2009.10.006

[B59] Sugden MC Zariwala MG Holness MJ PPARs and the orchestration of metabolic fuel selection Pharmacol Res 2009 60 3 141 150 10.1016/j.phrs.2009.03.014 19646653

[B60] Yoon M The role of PPARalpha in lipid metabolism and obesity: focusing on the effects of estrogen on PPARalpha actions Pharmacol Res 2009 60 3 151 159 10.1016/j.phrs.2009.02.004 19646654

[B61] Vazquez-Vela ME Torres N Tovar AR White adipose tissue as endocrine organ and its role in obesity Arch Med Res 2008 39 8 715 728 10.1016/j.arcmed.2008.09.005 18996284

[B62] Lefterova MI Lazar MA New developments in adipogenesis Trends Endocrinol Metab 2009 20 3 107 114 10.1016/j.tem.2008.11.005 19269847

[B63] Dahlman I Nilsson M Jiao H Hoffstedt J Lindgren CM Humphreys K Kere J Gustafsson JA Arner P Dahlman-Wright K Liver X receptor gene polymorphisms and adipose tissue expression levels in obesity Pharmacogenet Genomics 2006 16 12 881 889 10.1097/01.fpc.0000236334.49422.48 17108812

[B64] Das UN Obesity: genes, brain, gut, and environment Nutrition 2010 26 5 459 473 10.1016/j.nut.2009.09.020 20022465

[B65] Swartz-Basile DA Lu D Basile DP Graewin SJ Al-Azzawi H Kiely JM Mathur A Yancey K Pitt HA Leptin regulates gallbladder genes related to absorption and secretion Am J Physiol Gastrointest Liver Physiol 2007 293 1 G84 90 10.1152/ajpgi.00389.2006 17463181

[B66] Schmidt C Gonzaludo NP Strunk S Dahm S Schuchhardt J Kleinjung F Wuschke S Joost HG Al-Hasani H A meta-analysis of QTL for diabetes-related traits in rodents Physiol Genomics 2008 34 1 42 53 10.1152/physiolgenomics.00267.2007 18397992

[B67] Toro S Wegner J Muller M Westerfield M Varga ZM Identification of differentially expressed genes in the zebrafish hypothalamic-pituitary axis Gene Expr Patterns 2009 9 4 200 208 10.1016/j.gep.2008.12.007 19166982 PMC2804439

[B68] Westerfield M A guide for the laboratory use of zebrafish (Danio rerio) 2007 University of Oregon Press

[B69] Gentleman RC Carey VJ Bates DM Bolstad B Dettling M Dudoit S Ellis B Gautier L Ge Y Gentry J Bioconductor: open software development for computational biology and bioinformatics Genome Biol 2004 5 10 R80 10.1186/gb-2004-5-10-r80 15461798 PMC545600

