# Supplementary material for: Diet-induced obesity in zebrafish shares common pathophysiological pathways with mammalian obesity
Source: BMC Physiol. 2010 Oct 21;10:21. doi: 10.1186/1472-6793-10-21 (PMC2972245; doi:10.1186/1472-6793-10-21)
Supplement: Additional file 7 — Supplemental Figure S1. Validation of the differential gene expression by qPCR analysis. Total RNA was extracted from zebrafish visceral AT and qPCR analysis was performed to validate the differential expression identified by microarray analysis. Results represent means ± S.E.M. of each group. P-value was calculated by Student's t-test using OF1W (N = 6) vs. OF8W (N = 4). *p < 0.05. [file 1472-6793-10-21-S7.PDF]

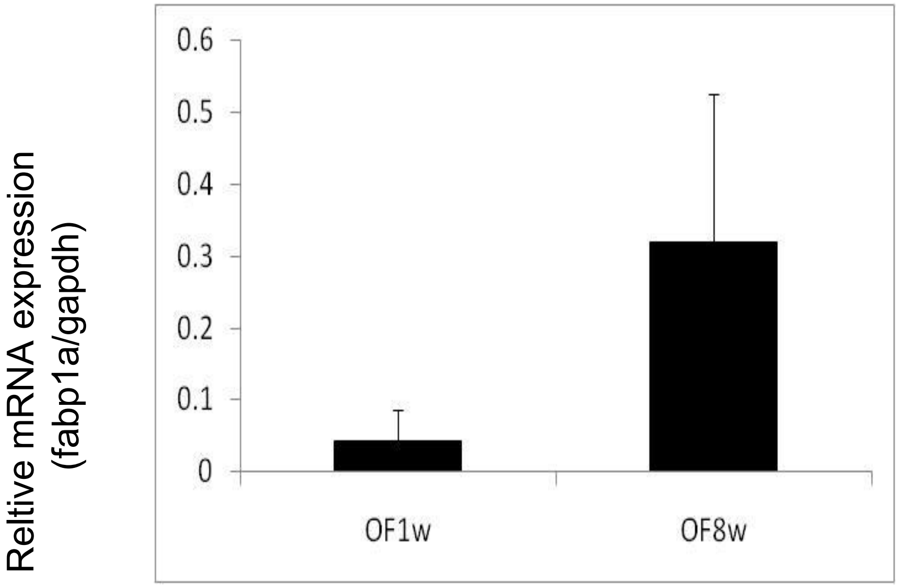
Reltive mRNA expression
(fabp1a/gapdh)
0.6
0.5
0.4
0.3
0.2
0.1
0
OF1w
OF8w

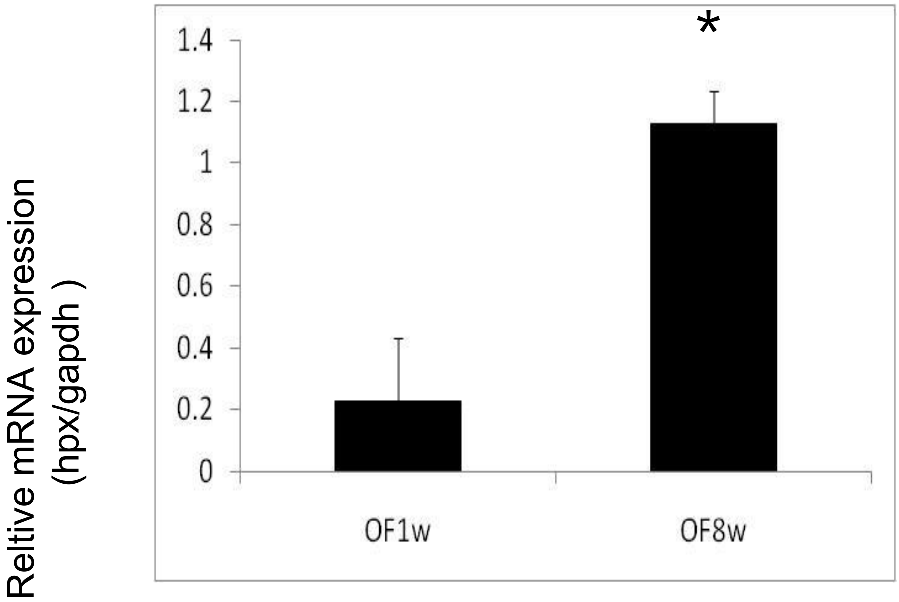
Reltive mRNA expression
(hpx/gapdh)
*
1.4
1.2
1
0.8
0.6
0.4
0.2
0
OF1w
OF8w

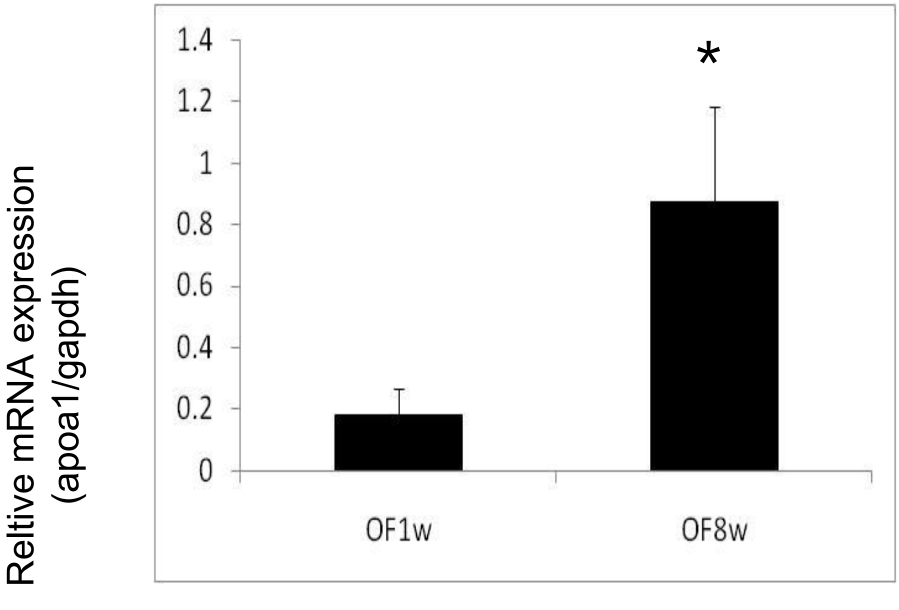
Reltive mRNA expression
(apoa1/gapdh)
*
1.4
1.2
1
0.8
0.6
0.4
0.2
0
OF1w
OF8w
